# Smartphone-Based Photoelectrochemical Immunoassay with Co_9_S_8_@ZnIn_2_S_4_ for Point-of-Care Diagnosis of Breast Cancer Biomarker

**DOI:** 10.34133/2022/9831521

**Published:** 2022-08-18

**Authors:** Ruijin Zeng, Yuxuan Li, Yanli Li, Qing Wan, Zhisheng Huang, Zhenli Qiu, Dianping Tang

**Affiliations:** ^1^Key Laboratory of Analytical Science for Food Safety and Biology (MOE & Fujian Province), State Key Laboratory of Photocatalysis on Energy and Environment, Department of Chemistry, Fuzhou University, Fuzhou 350108, China; ^2^School of Electronics and Information Engineering, Hubei University of Science and Technology, Xianning 437100, China; ^3^College of Materials and Chemical Engineering, Minjiang University, Fuzhou 350108, China

## Abstract

Photoelectrochemical immunoassays incorporating specific antigen-antibody recognition reactions with the photon-electron conversion capabilities of photocatalysts have been developed for biomarker detection, but most involve bulky and expensive equipment and are unsuitable for point-of-care testing. Herein, a portable smartphone-based photoelectrochemical immunoassay was innovatively designed for the on-site detection of breast cancer biomarkers (human epidermal growth factor receptor 2; HER2). The system consists of a split-type immunoassay mode, disposable screen-printed electrode covered with hierarchical Co_9_S_8_@ZnIn_2_S_4_ heterostructures, an integrated circuit board, and a Bluetooth smartphone equipped with a specially designed app. Using alkaline phosphatase (ALP) catalytic strategy to in situ generate ascorbic acid (AA) for electron-donating toward Co_9_S_8_@ZnIn_2_S_4_ heterostructures, an immunoreaction was successfully constructed for the HER2 detection in the real sample due to the positive correlation of the photocurrent signal to electron donor concentration. Differential charge density indicates that the formation of Co_9_S_8_@ZnIn_2_S_4_ heterojunction can facilitate the flow of charges in the interface and enhance the photocurrent of the composite. More importantly, the measured photocurrent signal can be wirelessly transmitted to the software and displayed on the smartphone screen to obtain the corresponding HER2 concentration value. The photocurrent values linearly with the logarithm of HER2 concentrations range spanned from 0.01 ng/mL to 10 ng/mL with a detection limit of 3.5 pg/mL. Impressively, the clinical serum specimen results obtained by the proposed method and the wireless sensing device are in good agreement with the enzyme-linked immunosorbent assay (ELISA).

## 1. Introduction

Photoelectrochemical (PEC) immunoassays can convert biomolecular recognition process into readable photocurrent through electrons or holes transfer between photoactive species and electrodes surface, which has a lower limit of detection (LOD) and reduced background signal than traditional electrochemical strategy [1–4]. However, expensive and complicated electrochemical workstations and corresponding accessories are required to measure weak photocurrents in traditional PEC detections, which complicates the instruments and restricts the development of PEC detection systems toward low cost and portability to a certain extent. Therefore, the strategy of replacing electrochemical workstations and corresponding accessories with miniature devices is an important challenge, which can accelerate the translation of PEC bioanalysis from the laboratory to real life. To overcome the above problems, our research group also proposed some portable devices to improve the portability of PEC signal reading. For instance, Shu et al. devised power-free PEC immunoassays for the detection of prostate specific antigen with a portable digital multimeter [5]. In particular, the change of the instantaneous current value recorded by the digital multimeter shows a good correlation with the concentration of the prostate specific antigen, which greatly improved the portability of the PEC sensor system. Furthermore, Yu et al. established a system for the convenient and sensitive PEC detection of biomarkers using a commercialized LED flashlight and digital multimeter as the readout equipment [6]. The available LED flashlight can replace the xenon lamp unit equipped on the workstation, making all PEC system construction, inspection, and reading out of large equipment. Despite the huge leap mentioned above, data based on digital multimeter readings cannot be stored and further analyzed. In addition, the reading process cannot be displayed in real-time similar to the workstation interface. Recently, with the upgrading of application software, the smartphone plays a pivotal role in the fields of molecular diagnosis, food safety, biosecurity, and environmental monitoring [7–10]. Smartphones provide many excellent functions for mobile modes, such as touch screens, wireless transmission, programmability, and data storage, which stimulate the upsurge of peripheral devices related to smartphones. Meanwhile, based on the development of integrated circuits and microelectromechanical technologies, many highly integrated miniaturized devices and tiny-sized planar substrates (screen-printed electrodes; SPE) have been constructed to replace sophisticated electrochemical workstations and electrodes. Therefore, combining smartphones with integrated circuits and commercialized microelectrodes to construct a portable PEC sensing instrument is of great significance for expanding the application of PEC to practical clinical applications.

Actually, the preparation of a photocatalyst with a stable and highly sensitive analyte response is the core of the PEC immunoassays. Among various metal sulfide semiconductors, ZnIn_2_S_4_ has been shown to attract more and more attention in PEC sensors due to its suitable band gap, unique electronic, optical properties, and tunable morphological structure [11–13]. However, the defects of rapid recombination of photogenerated carriers, insufficient active sites, and severe photocorrosion of pure ZnIn_2_S_4_ limit the application in PEC immunoassays. Alternatively, rational coupling of ZnIn_2_S_4_ with appropriate band structure can effectively accelerate the separation and transfer of photoexcited charges, while also improving photostability and light-harvesting ability. As a potential photocatalyst, Co_9_S_8_ has the advantages of a narrow band gap and efficient charge transfer. Some works have combined the Co_9_S_8_ cocatalyst with other semiconductor materials (CdS or ZnS) for the construction of efficient PEC systems [14, 15]. Therefore, loading the Co_9_S_8_ cocatalyst on ZnIn_2_S_4_ nanosheets can promote the separation of photogenerated electron-hole pairs and obtain stable photocurrent signals.

**Scheme 1:**
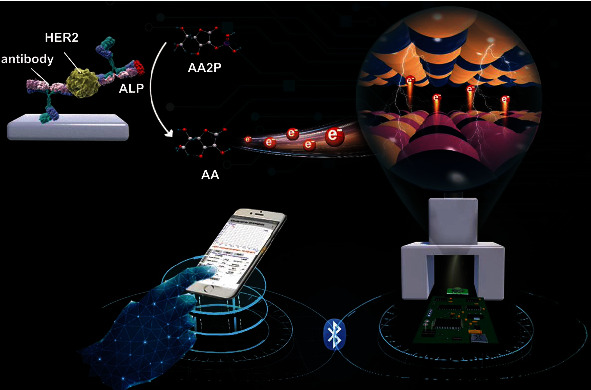
Schematic illustration of a smartphone-based photoelectrochemical immunoassay for the detection of HER2.

Breast cancer is a global health problem that seriously threatens women's physical health and is one of the malignant tumors with the highest incidence in women. The human epidermal growth factor receptor (HER2) gene, also known as CerbB-2, is overexpressed in 25-30% of breast cancers, making it the most common marker of breast cancer malignancy [16–18]. Accumulating evidence suggests that HER2 concentrations in the blood of breast cancer patients range from 15-75 ng/mL. Therefore, it is necessary to develop a highly accurate, portable, and specific assay to diagnose the concentration of HER2. Herein, a split-type and portable PEC immunosensing platform with smartphone readout coupling with highly integrated miniaturized devices and Co_9_S_8_@ZnIn_2_S_4_-modified SPE was designed for flexible detection of HER2 (Scheme 1). Specifically, Co_9_S_8_@ZnIn_2_S_4_ modification on SPE as a photoactive material realizes photoelectric conversion and exhibits significantly enhanced PEC performance with good photocurrent response to ascorbic acid. Accompanied by the specific antigen-antibody reaction and the corresponding ALP (alkaline phosphatase) catalyzes the hydrolysis of ascorbic acid 2-phosphate (AA2P) to generate ascorbic acid, the released ascorbic acid can effectively trap holes and inhibit electron-hole recombination, thereby triggering the photocurrent amplification of Co_9_S_8_@ZnIn_2_S_4_. With the help of a hand-held miniaturized circuit board, the photocurrent corresponding to different concentrations of HER2 can be measured and transmitted to a smartphone for display *via* Bluetooth. This work combines the sensitivity of PEC measurements with the portability of tiny circuit boards and smartphones, offering smaller sample volumes and more portable operation than conventional PEC assays.

## 2. Results and Discussion

### 2.1. Characterization of Co(CO_3_)_0.35_Cl_0.20_(OH)_1.10_, Co_9_S_8_, and Co_9_S_8_@ZnIn_2_S_4_

**Figure 1:**
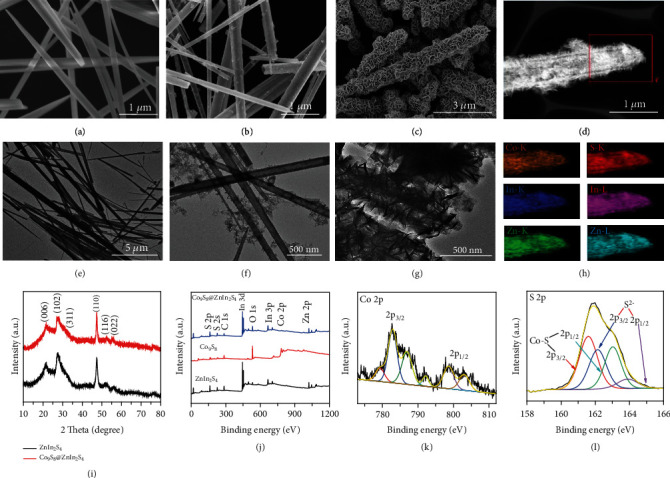
(a, e) SEM and TEM images of Co(CO_3_)_0.35_Cl_0.20_(OH)_1.10_. (b, f) SEM and TEM images of Co_9_S_8_. (c, g) SEM and TEM images of Co_9_S_8_@ZnIn_2_S_4_. (d) HAADF-STEM and (h) elemental mapping of Co_9_S_8_@ZnIn_2_S_4_. (i) XRD patterns of ZnIn_2_S_4_ and Co_9_S_8_@ZnIn_2_S_4_. (i) XPS survey spectra of Co_9_S_8_, ZnIn_2_S_4_, and Co_9_S_8_@ZnIn_2_S_4_; high-resolution XPS spectra of (k) Co 2p and (l) S 2p.

Figure S1 shows a brief schematic diagram of the synthesis process of hierarchical Co_9_S_8_@ZnIn_2_S_4_ tubular heterostructures. Co(CO_3_)_0.35_Cl_0.20_(OH)_1.10_ is a sacrificial template-directed route for the preparation of hollow Co_9_S_8_ nanotubes *via* the Kirkendall effect under hydrothermal conditions [19]. Subsequently, ZnIn_2_S_4_ nanosheets are grown on the surface of hollow Co_9_S_8_ nanotubes to form Co_9_S_8_@ZnIn_2_S_4_ heterostructures. The corresponding specific morphologies of the as-prepared samples (Co(CO_3_)_0.35_Cl_0.20_(OH)_1.10_, Co_9_S_8_, and Co_9_S_8_@ZnIn_2_S_4_) are characterized by scanning electron microscopy (SEM) and transmission electron microscopy (TEM). Figures 1(a) and 1(e) show that Co(CO_3_)_0.35_Cl_0.20_(OH)_1.10_ is a needle-like nanorod structure with a diameter of 80-220 nm and a length of several micrometers. When Co(CO_3_)_0.35_Cl_0.20_(OH)_1.10_ nanorods are treated in Na_2_S solution, the obtained Co_9_S_8_ still had a rod-like structure (Figure 1(b)). It is worth noting that the ends of Co_9_S_8_ nanotubes are broken openings from the SEM image, which is further confirmed by the hollow structure in the TEM image (Figure 1(f)). Subsequently, ultrathin ZnIn_2_S_4_ nanosheets (Figure S2) are grown on the surface of Co_9_S_8_ nanotubes using a low-temperature solvothermal treatment method. As shown in Figures 1(c) and 1(g), ZnIn_2_S_4_ nanosheets are uniformly and densely coated on the surface of Co_9_S_8_ nanotubes, forming Co_9_S_8_@ZnIn_2_S_4_ tubular heterostructure. The heterojunction between the ZnIn_2_S_4_ nanosheets and Co_9_S_8_ nanotubes is shown in high-resolution TEM (HRTEM) images (Figure S3). The interplanar spacings of 0.322 nm and 0.281 nm are clearly visible in the HRTEM image, which can be assigned to (102) planes of ZnIn_2_S_4_ and (222) planes of Co_9_S_8_, respectively [20, 21]. Moreover, high-angle annular darkfield scanning TEM (HAADF-STEM; Figure 1(d)) and corresponding selected area elemental mapping (Figure 1(h)) of Co_9_S_8_@ZnIn_2_S_4_ show the good distribution of S, Zn, In, and Co elements. The above electron microscopy results indicate that a tight and uniform heterojunction between Co_9_S_8_ nanotubes and ZnIn_2_S_4_ nanosheets can be successfully constructed through the designed hydrothermal route. Besides, the ZnIn_2_S_4_ displays five primary diffraction peaks in X-ray diffraction (XRD) at 21.61°, 27.84°, 47.42°, 52.26°, and 55.06°, which are consistent with hexagonal ZnIn_2_S_4_ (JCPDS no. 65-2023) (Figure 1(i)). As expected, the prepared Co_9_S_8_@ZnIn_2_S_4_ heterojunction additionally exhibited a diffraction peak (311) belonging to Co_9_S_8_ near 32.15°. As shown from the X-ray photoelectron spectroscopy (XPS) survey spectrum in Figure 1(j), all elements' valences (S 2p, 2 s; In 3d, 3p, Zn 2p, and Co 2p) related to Co_9_S_8_ and ZnIn_2_S_4_ can be observed in Co_9_S_8_@ZnIn_2_S_4_, which is consistent with the results of elemental mapping. The high-resolution Co 2p spectrum in Figure 1(k) consists of two spin-orbit doublets, where the first doublet at 779.32 eV and 782.85 eV and the second doublet at 792.17 eV and 798.61 eV correspond to Co 2p_3/2_ and Co 2p_1/2_, indicating the coexistence of Co^2+^ and Co^3+^ in the Co_9_S_8_@ZnIn_2_S_4_ [22]. Notably, the binding energy of Co 2p is shifted compared to pure Co_9_S_8_ (Figure S4), indicating a strong interfacial interaction between Co_9_S_8_ and ZnIn_2_S_4_. The peaks with binding energies around 163.89 eV and 163.03 eV correspond to S 2p_1/2_ and S 2p_3/2_ of the S^2−^ (ZnIn_2_S_4_), while the other two peaks at 162.14 eV and 161.59 eV are attributed to S 2p_1/2_ and S 2p_3/2_ of Co-S (Co_9_S_8_) (Figure 1(l)) [23, 24]. High-resolution XPS spectroscopy verified the existence of trivalent indium and divalent zinc in the nanocomposites (Figure S5) [25, 26]. Finally, the Brunauer-Emmett-Teller (BET) surface area of Co_9_S_8_, ZnIn_2_S_4_, and Co_9_S_8_@ZnIn_2_S_4_ are measured by a nitrogen gas adsorption-desorption isotherm. The calculated BET surface area (49.9350 m^2^/g) of Co_9_S_8_@ZnIn_2_S_4_ is larger than that of Co_9_S_8_ (6.8025 m^2^/g) and ZnIn_2_S_4_ (33.1675 m^2^/g) (Figure S6A-C), indicating that the uniform loading of ZnIn_2_S_4_ nanosheets can effectively increase the specific surface area of the Co_9_S_8_@ZnIn_2_S_4_ composite. Such a large specific surface area can provide more active sites for catalytic reactions. Meanwhile, the corresponding pore size distribution curves indicated the existence of mesopores, which would facilitate mass transfer in heterogeneous catalysis (Figure S6D-F).

### 2.2. Charge Carrier Behaviors and DFT Calculation of Co_9_S_8_@ZnIn_2_S_4_

**Figure 2:**
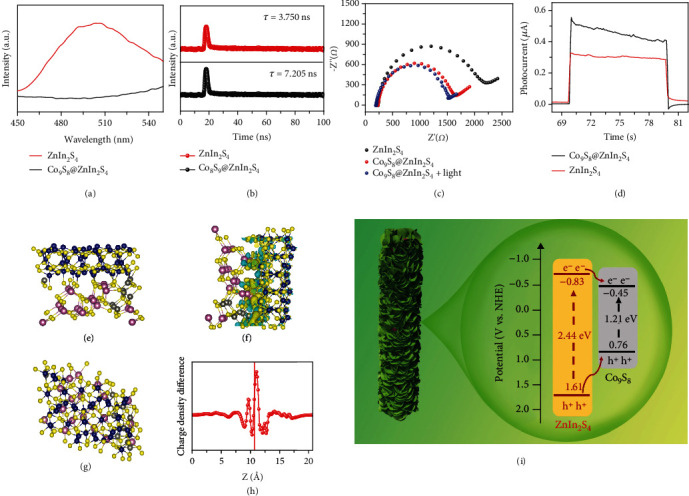
(a) Steady-state photoluminescence spectra. (b) Time-resolved photoluminescence decay. (d) Periodic on/off photocurrent responses of ZnIn_2_S_4_ and Co_9_S_8_@ZnIn_2_S_4_. (c) Electrochemical impedance spectra Nyquist plots of ZnIn_2_S_4_, Co_9_S_8_@ZnIn_2_S_4_, and Co_9_S_8_@ZnIn_2_S_4_ under illumination. (e, f) optimized structural model (top view and side view) of the Co_9_S_8_@ZnIn_2_S_4_. (g, h) The charge density distribution of Co_9_S_8_@ZnIn_2_S_4_. (i) transfer process of the photogenerated electrons and holes in the Co_9_S_8_@ZnIn_2_S_4_ heterostructure.

As mentioned above, since the readout of photocurrent is an important part of constructing a portable PEC immunoassay, the optical/photoelectrochemical properties of ZnIn_2_S_4_ before and after Co_9_S_8_ loading were further tested. The absorption properties and deduce the band gaps of Co_9_S_8_, ZnIn_2_S_4_, and Co_9_S_8_@ZnIn_2_S_4_ are characterized by UV-vis diffuse reflectance spectroscopy (DRS). ZnIn_2_S_4_ exhibits an absorption edge of around 520 nm, while Co_9_S_8_ shows a very broad absorption edge between 200 to 800 nm (Figure S7A). Compared with ZnIn_2_S_4_, Co_9_S_8_@ZnIn_2_S_4_ has an increased absorption band edge in the visible region, indicating that the sensitization of Co_9_S_8_ extends the visible light absorption properties of ZnIn_2_S_4_. The Tauc plot (Figure S7B) corresponding to DRS calculated the band gap values of ZnIn_2_S_4_ and Co_9_S_8_ to be 2.44 eV and 1.21 eV, respectively. The charge transfer kinetics and the lifetimes of ZnIn_2_S_4_ and Co_9_S_8_@ZnIn_2_S_4_ were further investigated by photoluminescence and time-resolved photoluminescence. The steady-state photoluminescence spectra (Figure 2(a)) indicate that the emission peak intensity of Co_9_S_8_@ZnIn_2_S_4_ is significantly lower than that of ZnIn_2_S_4_, indicating that the prohibited recombination of photo-excited charges of Co_9_S_8_@ZnIn_2_S_4_. Meanwhile, the time-resolved photoluminescence decay spectra and the corresponding exponential decay kinetics function results show that the average emission lifetime of Co_9_S_8_@ZnIn_2_S_4_ (*τ* = 7.205 ns; *τ*_1_ = 0.5363 ns, *A*_1_ = 68.12%, *τ*_2_ = 21.454 ns, *A*_2_ = 31.88%) is longer than that of ZnIn_2_S_4_ (*τ* = 3.750 ns; *τ*_1_ = 0.7730 ns, *A*_1_ = 40.68%, *τ*_2_ = 5.7915 ns, and *A*_2_ = 59.32%) (Figure 2(b)), illustrating the possible existence of more high-speed charge transfer channels between Co_9_S_8_ and ZnIn_2_S_4_. Comprehensive electrochemical impedance spectra (Figure 2(c)) and photocurrent measurement (Figure 2(d)) demonstrate that Co_9_S_8_@ZnIn_2_S_4_ had a smaller semicircle in Nyquist plots under light and dark conditions and higher photocurrent intensity than ZnIn_2_S_4_. The above characterization results collectively demonstrated that the combination of Co_9_S_8_ and ZnIn_2_S_4_ has a better light absorption property and the ability to separate and transfer photoexcited carriers.

To help elucidate the effect of photocurrent enhancement upon ZnIn_2_S_4_ loading with Co_9_S_8_, a density functional theory (DFT) approach was employed. From the HRTEM results, we constructed the interface between the (102) facet of ZnIn_2_S_4_ and the (222) facet of Co_9_S_8_. We constructed a matching structure of Co_9_S_8_(222)/ZnIn_2_S_4_(102) (optimized structures in Figures 2(e) and 2(f)) and further analyzed the electron density distribution at the heterojunction interface. The corresponding simulated electron density distribution shows that the accumulated electrons are mainly distributed on the Co_9_S_8_ (222) face and the electron-deficient on the ZnIn_2_S_4_ (102) interface, confirming the strong electron transfer from ZnIn_2_S_4_ (102) face to Co_9_S_8_ (222) face at the heterojunction interface (Figures 2(g) and 2(h)). Therefore, the formation of Co_9_S_8_@ZnIn_2_S_4_ heterojunction is beneficial to the separation of photogenerated electrons and realizes the amplification of photocurrent. To confirm the electron transfer path, we further estimate the conduction band (CB) and valence band (VB) positions. ZnIn_2_S_4_ and Co_9_S_8_ exhibit typical features of n-type semiconductors due to the positive slope of the Mott-Schottky plots, and the derived flat band potentials (E_fb_) is approximate -1.03 V and -0.65 V, respectively (Figure S8). Therefore, the *E*_*fb*_ of ZnIn_2_S_4_ and Co_9_S_8_ is calculated to be -0.83 V and -0.45 V, respectively. Considering that the value of the *E*_*fb*_ of n-type semiconductors is approximately equal to the value of the conduction band potential (*E*_*CB*_), the *E*_*CB*_ of ZnIn_2_S_4_ and Co_9_S_8_ is -0.83 V and -0.45 V, respectively. Combining the above band gap value and the formula *E*_*g*_ = *E*_*VB*_ − *E*_*CB*_, the valence band potentials (*E*_*VB*_) of ZnIn_2_S_4_ and Co_9_S_8_ are calculated to be 1.61 V and 0.76 V, respectively. Based on these experimental and theoretical results, we propose a possible working mechanism for Co_9_S_8_@ZnIn_2_S_4_ heterostructure (Figure 2(i)). In the type-I heterostructure of Co_9_S_8_@ZnIn_2_S_4_, the photogenerated CB electrons of ZnIn_2_S_4_ can rapidly migrate to the CB of Co_9_S_8_ through the heterojunction interface due to the more negative CB position of ZnIn_2_S_4_. Therefore, the photogenerated electron-hole pairs are effectively separated in the Co_9_S_8_@ZnIn_2_S_4_ heterostructure.

### 2.3. Analytical Performance of the Smartphone-Based PEC Immunoassay

**Figure 3:**
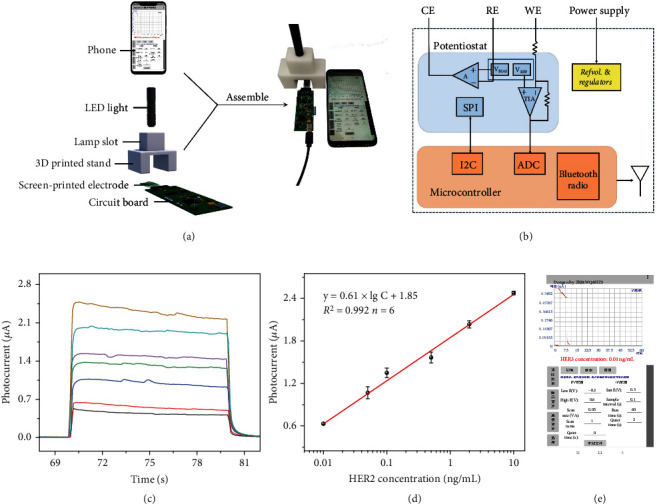
(a) Exploded view of a smartphone-based PEC sensing system for portable detection of HER2, including smartphone, LED light, Co_9_S_8_@ZnIn_2_S_4_-modified SPE, and electronic readout circuit. (b) Block diagram of electronic readout circuit. (c) Photocurrent-time curves of Co_9_S_8_@ZnIn_2_S_4_-modified SPE toward HER2 with different concentrations. (d) Corresponding calibration curve between the photocurrent intensity and HER2 concentrations. (e) Smartphone screen of photocurrent measurements and HER2 concentrations converted from linear equations.

To realize the PEC immunoassay according to the predetermined strategy, we first explored the photocurrent response of Co_9_S_8_@ZnIn_2_S_4_-modified SPE toward ascorbic acid. As shown in Figure S9, the photocurrents of Co_9_S_8_@ZnIn_2_S_4_-modified SPE reacted with ascorbic acid concentrations of 0 nM and 500 nM are 0.514 *μ*A and 1.569 *μ*A, respectively. The roughly 3.05-fold increase in photocurrent indicates that Co_9_S_8_@ZnIn_2_S_4_-modified SPE has a good photocurrent response to trace amounts of ascorbic acid. To make the photocurrent signal detection and reading more portable, we designed a detection system including LED light, 3D printed stand, a self-designed integrated circuit, and a smartphone. Photographs of the part assembly process and assembly results are shown in Figure 3(a). The detail operating process of the system is shown in Figure 3(b). Specifically, the photocurrent signal obtained from the PEC sensor is converted into a digital signal through an analog-to-digital converter. Meanwhile, the data displayed on the smartphone screen can be exported for further analysis. The constructed PEC portable immunoassay was adopted to detect HER2 standards at various concentrations. With increasing HER2 concentration, more ascorbic acid was produced in the detection solution, increasing the photocurrent intensity of Co_9_S_8_@ZnIn_2_S_4_-modified SPE (Figure 3(c)). A good linear correlation was formed between the response photocurrent and the logarithm of the HER2 concentration (Figure 3(d)). The associated regression equation is expressed as *I* (nA) = 0.61 × lgC_[HER2]_ + 1.85 (ng/mL) (*R*^2^ = 0.992, *n* = 6) with a LOD of 3.5 pg/mL (calculated at 3*σ*). Impressively, compared to other existing HER2 detection methods, the developed PEC immunoassay allows for a lower LOD while fully considering portability (Table S1). The smartphone app can read the photocurrent value and calculate the HER2 concentration value according to the corresponding linear regression equation, making the whole detection process more convenient and efficient (Figure 3(e)). Besides, the maximum relative standard deviations (RSDs, *n* = 3) were 4.11%, 5.21%, and 4.98% for intra-assays, and 7.36%, 8.95%, and 7.62% for interassays toward 0.01, 0.1, and 10 ng/mL of HER2, respectively, indicating satisfactory reproducibility. Furthermore, the selectivity of the immunoassay was essential for appraising the analytical performance of the designed smartphone-based portable PEC immunoassay (Figure S10). In the presence of 20 ng/mL IgG (immunoglobulin G), CEA (carcinoembryonic antigen), and BSA (bull serum albumin), no obvious interferential photocurrent of Co_9_S_8_@ZnIn_2_S_4_-modified SPE occurred compared with the blank sample. In contrast, the presence of HER2 (10 ng/mL), as well as the abovementioned interference, is able to induce an increase in photocurrent, indicating the excellent specificity of this system.

### 2.4. Detection of HER2 in Serum Samples and Evaluation of Method Accuracy

**Figure 4:**
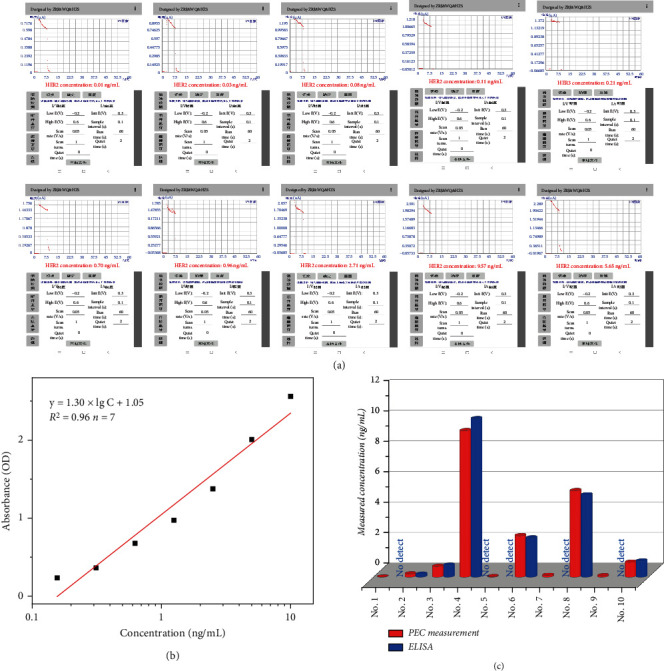
(a) Screenshot plots of the Android App for real sample detection using smartphone-based portable PEC immunoassay. (b) Concentration-absorbance linear equations associated with HER2 ELISA kits. (c) Comparison of HER2 concentrations measured with smartphone-based portable PEC immunoassay and those measured with a commercial ELISA for 10 clinical serum samples.

To evaluate the application of the proposed portable immunoassay in clinical diagnosis, we measured the concentration level of HER2 in 10 serum samples. As a reference, the same samples are also tested with commercial HER2 ELISA kit from Wuhan Cusabio Biotech. Inc. (Wuhan, China, https://www.cusabio.com/).  Figure 4(a) shows smartphone screenshots of photocurrents and corresponding HER2 concentration levels for different serum samples. Before comparison, a concentration-absorbance linear regression equation was obtained using different standard concentrations in the ELISA kit. The regression equation is expressed as *y* = 1.30 × lgC_[HER2]_ + 1.05 (ng/mL) (*R*^2^ = 0.96, *n* = 7, Figure 4(b)). The concentration results obtained from the two methods are summarized in Figure 4(c). Impressively, the proposed portable PEC immunoassay was able to detect lower abundances of HER2 compared to the ELISA method. On the basis of the values obtained by these two methods at higher concentrations, the accuracy of the method was evaluated by a regression equation, fitted as *y* = 1.013 *x* + 0.0118 (*R*^2^ = 0.9994, where *x* and *y* represent data from PEC immunoassay and HER2 ELISA kit). The slope and intercept in this regression equation are close to ideal “1” and “0,” respectively. Therefore, no significant differences were found between these two methods for analyzing serum samples, indicating that the proposed portable PEC immunoassay has good accuracy for the determination of target HER2 in human biological fluid samples.

## 3. Conclusion

In conclusion, this contribution devised a smartphone-based portable PEC immunoassay for the determination of breast cancer biomarkers (human epidermal growth factor receptor 2; HER2) by coupling with the Co_9_S_8_@ZnIn_2_S_4_-modified SPE system. In contrast to conventional PEC sensing techniques, the proposed strategy does not require the use of large-scale equipment during photocurrent testing and reading. An app running on a smartphone can measure the photocurrent in real-time and estimate the concentration of HER2 in the sample using a linear equation. If required, diagnostic results can be easily shared or transmitted to specific data platforms. Combining the high sensitivity of PEC technology and integrated circuit technology enables the proposed immunoassay to detect HER2 at 3.5 pg/mL, which is well below the clinical threshold. Given its outstanding detection performance and highly integrated circuit without additional electrochemical workstations and cumbersome control systems, the developed smartphone PEC immunoassay opens up a new approach toward the development of point-of-care detection, which is suitable for the clinical diagnosis, especially in resource-limited regions.

## 4. Materials and Methods

### 4.1. Preparation of Co_9_S_8_@ZnIn_2_S_4_-Modified Electrode

The electrodes used in this experiment were SPE in a commercial three-electrode configuration. The modification of SPE is carried out by dropping Co_9_S_8_@ZnIn_2_S_4_ solution (10 *μ*L, 1.5 mg/mL, ultrasound 5 min) over the working electrode surface. The Co_9_S_8_@ZnIn_2_S_4_ solution was dried on SPE at 65°C for 1 h to ensure the completion of the drying process. The Co_9_S_8_@ZnIn_2_S_4_-modified SPE was inserted into the circuit board and coupled with the LED light source to form a photoelectrochemical detection device.

### 4.2. Design of the PEC Detection System

Immunoreactions were performed according to the manufacturer's instructions. All samples and reagents should be left at room temperature for 0.5 h to return to room temperature before use. Initially, HER2 standard or sample solutions (100 *μ*L) of various concentrations were added to the well and incubated at 37°C for 2 h. After removing the liquid from each well, add biotin antibody (100 *μ*L) and incubate at 37°C for 1 h. Aspirate the liquid from each well and wash three times with washing buffer. After the final wash, completely blot the remaining liquid from the wells by inverting the wells on a clean paper. Subsequently, ALP was further loaded on the antibody by adding streptavidin-linked ALP solution (100 *μ*L, 20 nM) and incubating at 30°C for 1 h. After repeated washing of the wells four times, AA2P solution (50 *μ*L, 100 mM) was added and incubated at 37°C for 1 h. Finally, the reaction solution (50 *μ*L) and Na_2_SO_4_ solution (50 *μ*L, 0.2 M) are thoroughly mixed and dropped onto Co_9_S_8_@ZnIn_2_S_4_-modified SPE for photocurrent detection.

## Data Availability

The data used to support the findings of this study are available from the corresponding author upon request.

## Supplementary Materials

Supplementary Materials Figure S1: illustration of the fabrication process of hierarchical Co_9_S_8_@ZnIn_2_S_4_ tubular photocatalyst. Figure S2: SEM image of ZnIn_2_S_4_. Figure S3: HRTEM of Co_9_S_8_@ZnIn_2_S_4_. Figure S4: high-resolution XPS Co 2p spectra of Co_9_S_8_. Figure S5: high-resolution XPS spectra In 3d and Zn 2p of Co_9_S_8_@ZnIn_2_S_4_. Figure S6: N2 adsorption-desorption isotherms and pore size distribution curve of Co_9_S_8_, ZnIn_2_S_4_, and Co_9_S_8_@ZnIn_2_S_4_. Figure S7: DRS and Tauc plots of Co_9_S_8_, ZnIn_2_S_4_, and Co_9_S_8_@ZnIn_2_S_4_. Figure S8: Mott-Schottky plots of ZnIn_2_S_4_ andCo_9_S_8_ samples. Figure S9: photocurrent responses of the Co_9_S_8_@ZnIn_2_S_4_ containing 0 nM and 500 nM ascorbic acid. Figure S10: the anti-interference ability of proposed PEC immunoassay. Table S1: comparison of different HER2 detection methods on analytical properties.
