# Supplementary material for: Smartphone-Based Photoelectrochemical Immunoassay with Co9S8@ZnIn2S4 for Point-of-Care Diagnosis of Breast Cancer Biomarker
Source: Research (Wash D C). 2022 Aug 18;2022:9831521. doi: 10.34133/2022/9831521 (PMC9422330; doi:10.34133/2022/9831521)
Supplement: Supplementary Materials — Figure S1: illustration of the fabrication process of hierarchical Co9S8@ZnIn2S4 tubular photocatalyst. Figure S2: SEM image of ZnIn2S4. Figure S3: HRTEM of Co9S8@ZnIn2S4. Figure S4: high-resolution XPS Co 2p spectra of Co9S8. Figure S5: high-resolution XPS spectra In 3d and Zn 2p of Co9S8@ZnIn2S4. Figure S6: N2 adsorption-desorption isotherms and pore size distribution curve of Co9S8, ZnIn2S4, and Co9S8@ZnIn2S4. Figure S7: DRS and Tauc plots of Co9S8, ZnIn2S4, and Co9S8@ZnIn2S4. Figure S8: Mott-Schottky plots of ZnIn2S4 andCo9S8 samples. Figure S9: photocurrent responses of the Co9S8@ZnIn2S4 containing 0 nM and 500 nM ascorbic acid. Figure S10: the anti-interference ability of proposed PEC immunoassay. Table S1: comparison of different HER2 detection methods on analytical properties. [file 9831521.f1.docx]

**SUPPORTING INFORMATION**

**Smartphone-Based Photoelectrochemical Immunoassay with Co_9_S_8_@ZnIn_2_S_4_ for** **Point-of-care Diagnosis of Breast Cancer Biomarker**

Ruijin Zeng,^1,§^ Yuxuan Li,^1,§^ Yanli Li,^1^ Qing Wan,^2^ Zhisheng Huang,^2^ Zhenli Qiu,^3*^ and Dianping Tang^1^*

^1^Key Laboratory of Analytical Science for Food Safety and Biology (MOE & Fujian Province), State Key Laboratory of Photocatalysis on Energy and Environment, Department of Chemistry, Fuzhou University, Fuzhou, 350108, China.

^2^School of Electronics and Information Engineering. Hubei University of Science and Technology, Xianning 437100, China.

^3^College of Materials and Chemical Engineering, Minjiang University, Fuzhou 350108, China.

[^ξ^] These authors contributed equally to this work.

**EXPERIMENTAL SECTION**

**Material and Reagent.** Urea (CH_4_N_2_O, ≥ 99.5%), sodium sulfide (Na_2_S) cobalt chloride hexahydrate (CoCl_2_·6H_2_O, ≥ 98.0%), indium chloride tetrahydrate (InCl_3_·4H_2_O, 99.995% metals basis), thioacetamide (TAA, ≥ 99.0%), zinc chloride (ZnCl_2_, 99.95% metals basis), absolute ethanol (C_2_H_5_OH), glycerol (GL), hydrochloric acid (HCl, 37%), L-ascorbic acid 2-phosphate sesquimagnesium salt hydrate (AAP) and sodium sulfate (Na_2_SO_4_) were purchased from Aladdin Reagents (Shanghai, China) Co., Ltd. or Sinopharm Chemical Reagent Co., Ltd (Shanghai, China). Streptavidin-linked ALP was purchased from Promega Co. (Madison, WI, U.S.A.). HER2 ELISA Kit was purchased from Wuhan Cusabio Biotech. Co., Ltd.

**Preparation of** **Co_9_S_8_ nanotubes.** The Co_9_S_8_ nanotubes were synthesized by two-step hydrothermal methods. Co(CO_3_)_0.35_Cl_0.20_(OH)_1.10_ nanorod were first prepared as templates. Typically, CH_4_N_2_O (5 mmol) and CoCl_2_·6H_2_O (5 mmol) were dissolved into deionized water (40 mL) under the treatment of sonication. After sonication for 0.5 h, the obtained solution was then transferred into a Teflon-lined autoclave and maintained at 120 °C for 10 h. After the reaction, the pink precipitates were collected by centrifugation, rinsed with water and absolute ethanol three times, and dried in a vacuum. Afterward, the as-prepared Co(CO_3_)_0.35_Cl_0.20_(OH)_1.10_ precursor (0.11 g) was added into Na_2_S solution (5 mg/mL, 40 mL) in a Teflon liner under stirring for 60 min, then the liner was transferred into a stainless-steel autoclave and heated at 160 °C for 8 h in an oven. After cooling down to the room temperature naturally, the black products Co_9_S_8_ nanotubes were obtained by centrifugation, washed with absolute ethanol, and finally dried under vacuum.

**Preparation of Co_9_S_8_@ZnIn_2_S_4_ heterostructures.** The growth of ZnIn_2_S_4_ nanosheets on the surfaces of Co_9_S_8_ nanotubes was fabricated by a low-temperature solvothermal approach. In a typical experimental procedure, water (32 mL, adjusted by 0.5 M HCl) and glycerol (8 mL) were added into a 100 mL round-bottom flask under stirring to form the homogeneous solution. Next, ZnCl_2_ (272 mg), InCl_3_·4H_2_O (586 mg), and TAA (300 mg) were dissolved into the above solution by ultrasonication. Then, certain amounts of the Co_9_S_8_ nanotubes (21.2 mg) were added to the solution and the mixture was stirred for 30 min to realize the adsorption process. After that, the obtained mixture was put into an oil bath at 80 °C with continuous stirring for 2 h. Finally, the product was collected by centrifugation, washed with ethanol three times, and dried at 60 °C in a vacuum.

**Theoretical Calculation Method.** Our computations are based on the first-principles density functional theory (DFT) approach within the generalized gradient approximation (GGA) method. Vienna ab-initio simulation package (VASP) has been employed throughout the study. Here, the valence electronic states are expanded in a set of periodic plane waves, and the interaction amid core electrons and valence electrons is implemented through the projector augmented wave (PAW) method. Perdew-Burke-Ernzerhof (PBE) parametrized GGA functional are utilized to describe the exchange-correlation interactions. The wave functions were expanded into a basis set of plane waves with a kinetic energy cutoff of 450 eV. Ionic relaxations were carried out until the atomic forces were converged to 0.03 eV/Å. And the convergence threshold for self-consistence-field iteration was set at 10^−4^ eV.

**PARTIAL RESULTS AND DISCUSSION**


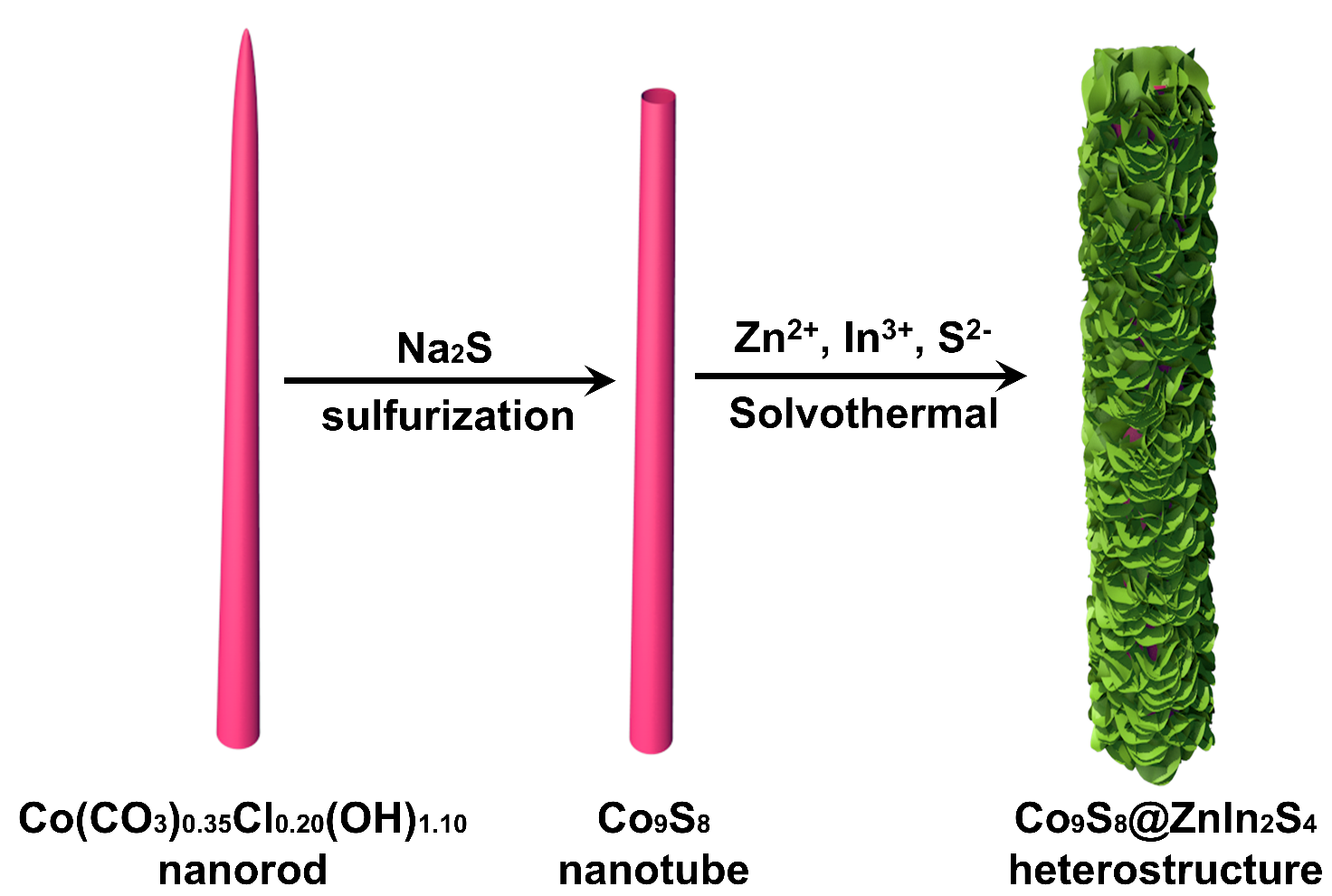


**Figure S1.** Illustration of the fabrication process of hierarchical Co_9_S_8_@ZnIn_2_S_4_ tubular photocatalyst.

**
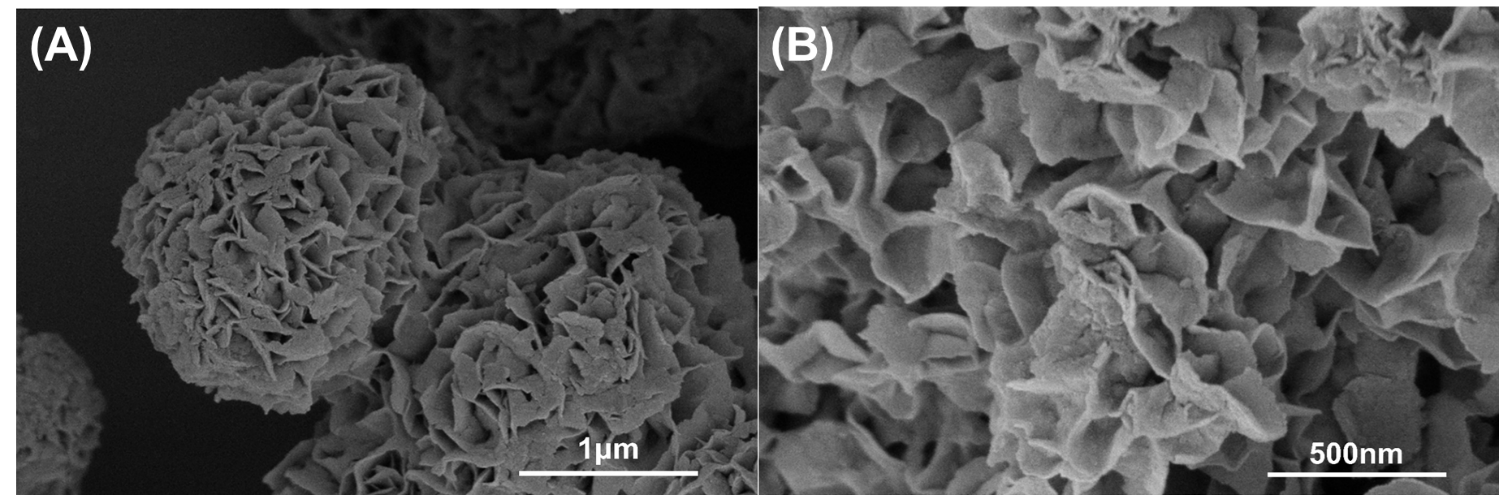
**

**Figure S2.** SEM images of ZnIn_2_S_4_.


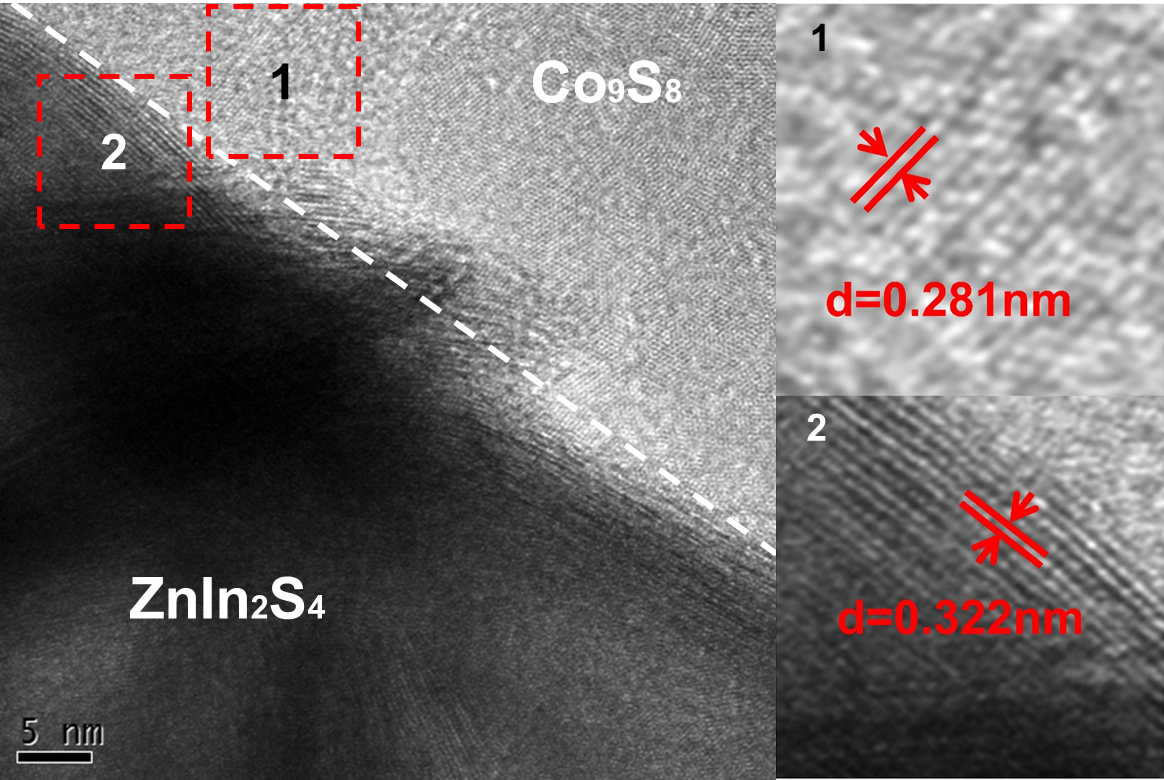


**Figure S3.** HRTEM of Co_9_S_8_@ZnIn_2_S_4_.


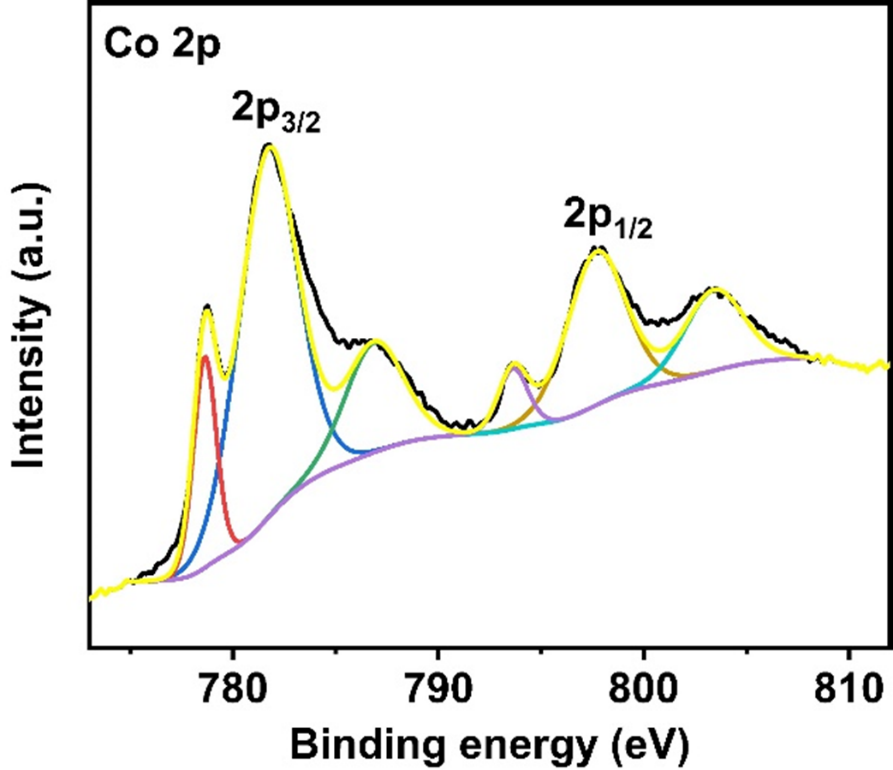


**Figure S4.** High-resolution XPS Co 2p spectra of Co_9_S_8_.

**
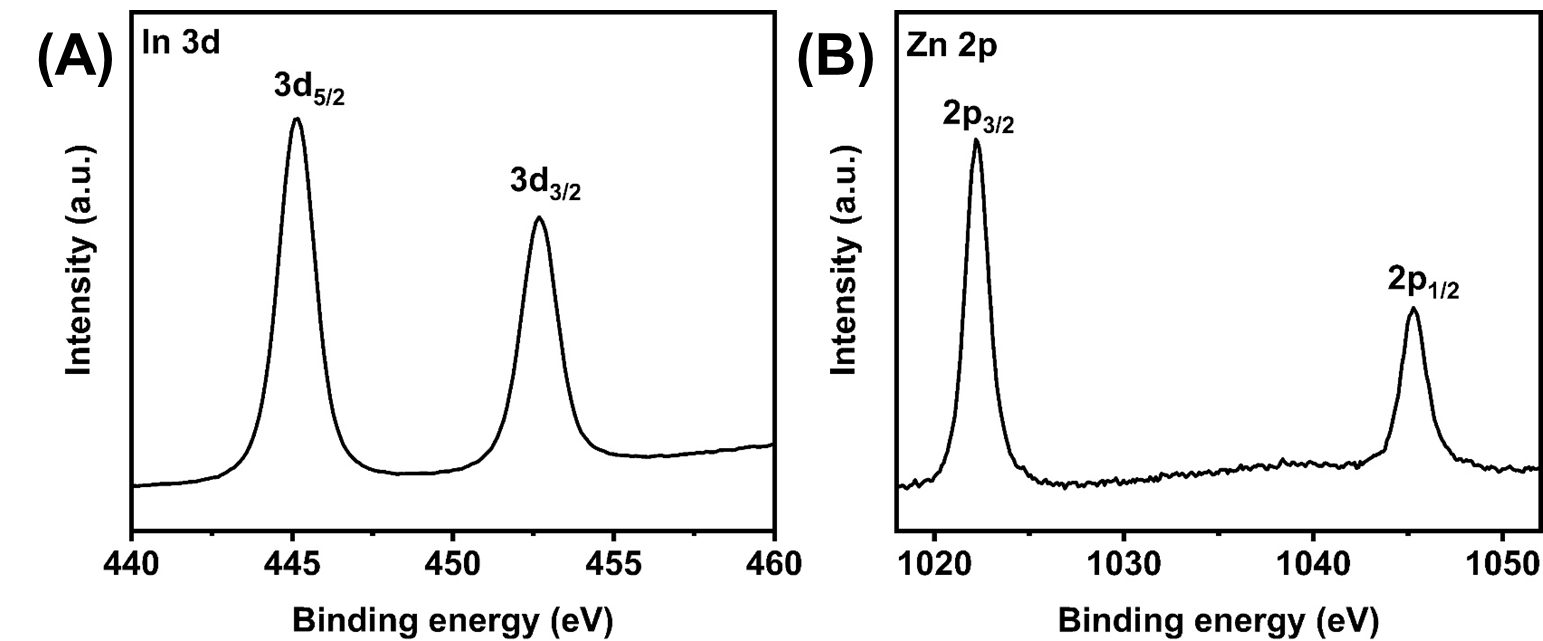
**

**Figure S5.** High-resolution XPS spectra In 3d (A) and Zn 2p (B) of Co_9_S_8_@ZnIn_2_S_4_. Meanwhile, the binding energies of In 3d_5/2_ and In 3d_3/2_ are 445.14 eV and 452.68 eV in the Co_9_S_8_@ZnIn_2_S_4_ (Figure S5A), which can be attributed to the trivalent indium state existing in the Co_9_S_8_@ZnIn_2_S_4_. Similarly, two separate peaks located at 1045.27 eV and 1022.23 eV are assigned to Zn 2p_1/2_ and Zn 2p_3/2_, respectively (Figure S5B), indicating the presence of divalent zinc.


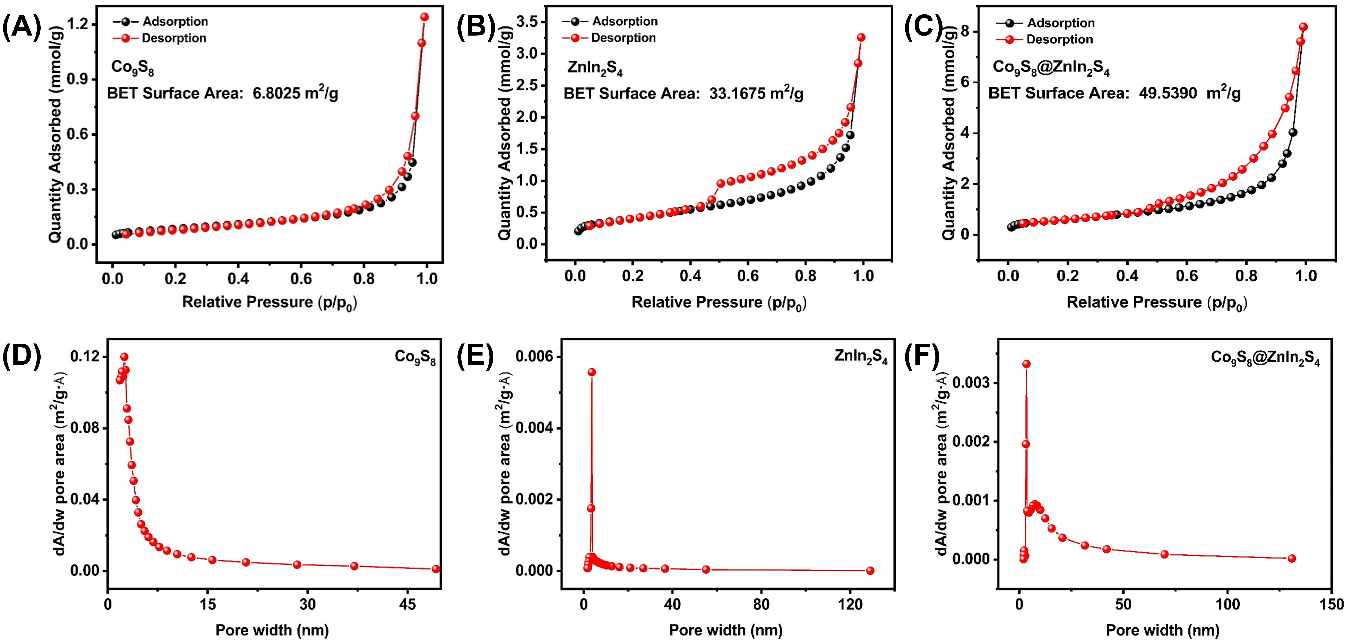


**Figure S6.** N_2_ adsorption-desorption isotherms of (A) Co_9_S_8_, (B) ZnIn_2_S_4_ and (C) Co_9_S_8_@ZnIn_2_S_4_; pore size distribution curve of (D) Co_9_S_8_, (E) ZnIn_2_S_4_ and (F) Co_9_S_8_@ZnIn_2_S_4_.


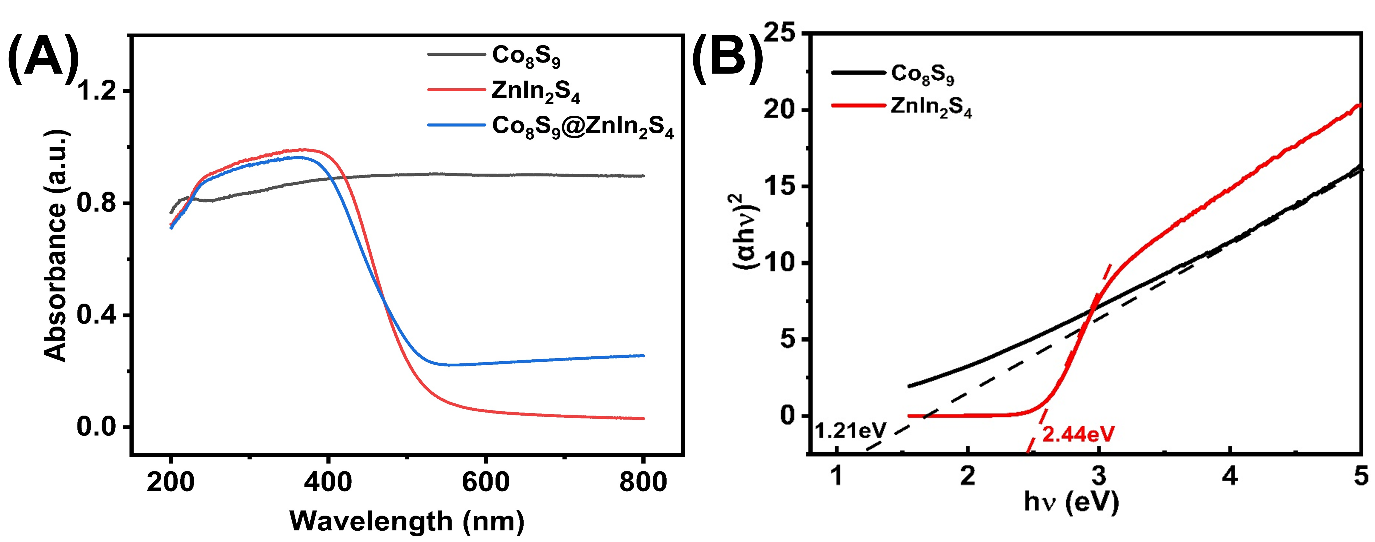


**Figure S7.** (A) DRS of Co_9_S_8_, ZnIn_2_S_4_ and Co_9_S_8_@ZnIn_2_S_4_; (B) Tauc plots of ZnIn_2_S_4_ and Co_9_S_8_.


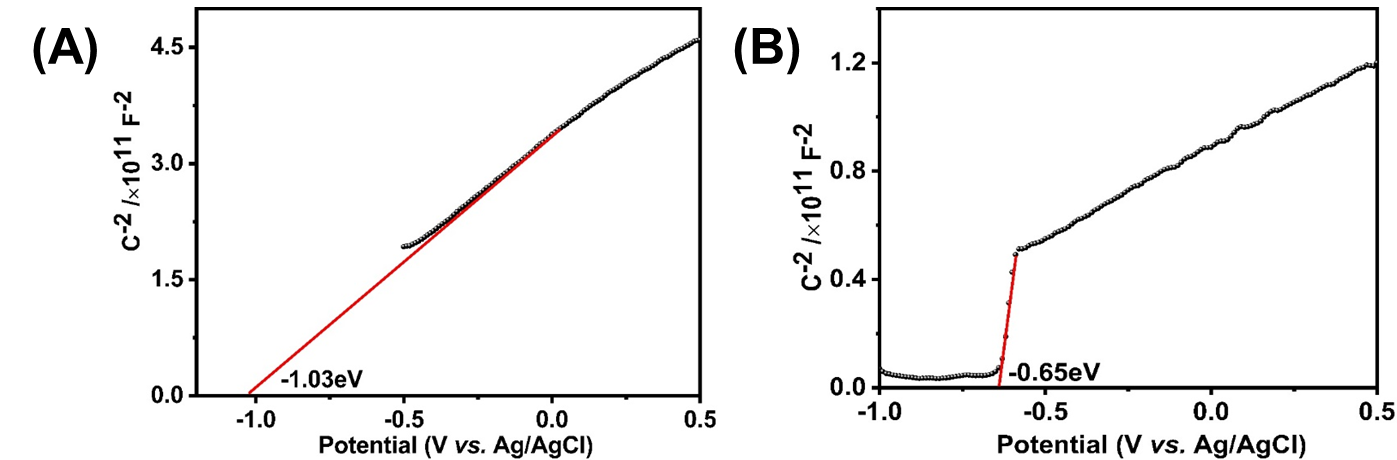


**Figure S8.** Mott-Schottky plots of (A) ZnIn_2_S_4_ and (B) Co_9_S_8_ samples.


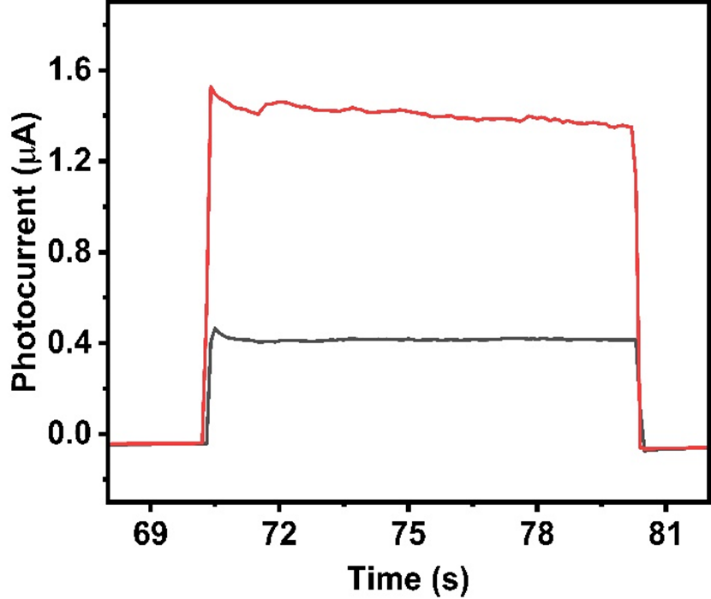


**Figure S9.** Photocurrent responses of the Co_9_S_8_@ZnIn_2_S_4_ containing 0 nM (black line) and 500 nM (red line) ascorbic acid.


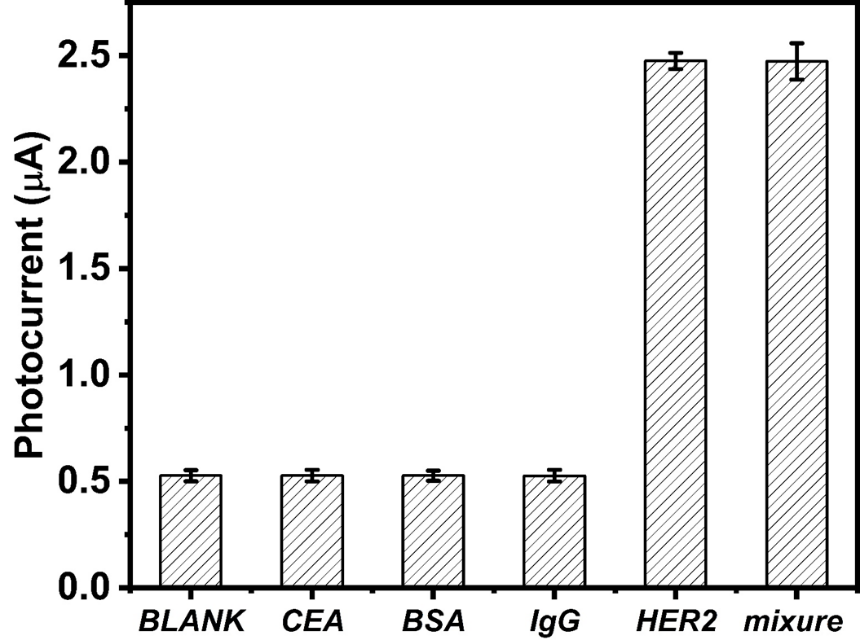


**Figure S10.** The anti-interference ability against 10 ng/mL HER2, 20 ng/mL CEA, 20 ng/mL BSA, 20 ng/mL IgG, the mixture containing the aforementioned analytes.

**Table S1. Comparison of Different HER2 Detection Methods on Analytical Properties.**

|  | | | |
| --- | --- | --- | --- |
| Method | Linear range | Limit of detection | Ref. |
| Electrochemical biosensor | 0.1 – 1000 ng/mL | 45 pg/mL | [1] |
| Multicolor immunosensor | 0.1 – 9.0 ng/mL | 0.05 ng/mL | [2] |
| Impedimetric immunosensor | 0.01 – 100 ng/mL | 0.01 ng/mL | [3] |
| Electrochemical biosensor | 0.1 – 100 ng/mL | 0.08 ng/mL | [4] |
| Photoelectrochemistry immunoassay | 0.01 – 10 ng/mL | 3.5 pg/mL | This work |

**REFERENCES**

[1] W. Wang, R. Han, M. Chen, X. Luo, "Antifouling peptide hydrogel based electrochemical biosensors for highly sensitive detection of cancer biomarker her2 in human serum," *Analytical Chemistry*, vol. 93, no. 19, pp. 7355-7361, 2021.

[2] Z. Wang, Q. Chen, Y. Zhong, X. Yu, Y. Wu, F. Fu, "A multicolor immunosensor for sensitive visual detection of breast cancer biomarker based on sensitive nadh-ascorbic-acid-mediated growth of gold nanobipyramids," *Analytical Chemistry*, vol. 92, no. 1, pp. 1534-1540, 2020.

[3] S. Sharma, J. Zapatero-Rodriguez, R. Saxena, R. O'Kennedy, S. Srivastava, "Ultrasensitive direct impedimetric immunosensor for detection of serum her2," *Biosensors and Bioelectronics*, vol. 106, pp. 78-85, 2018.

[4] D. Ou, D. Sun, X. Lin, Z. Liang, Y. Zhong, Z. Chen, "A dual-aptamer-based biosensor for specific detection of breast cancer biomarker her2 via flower-like nanozymes and DNA nanostructures," *Journal of Materials Chemistry B*, vol. 7, no. 23, pp. 3661-3669, 2019.
